# Changes in non-motor symptoms in patients with Parkinson's disease following COVID-19 pandemic restrictions: A systematic review

**DOI:** 10.3389/fpsyg.2022.939520

**Published:** 2022-07-22

**Authors:** Francesca Mameli, Eleonora Zirone, Benedetta Capetti, Denise Mellace, Roberta Ferrucci, Giulia Franco, Alessio Di Fonzo, Sergio Barbieri, Fabiana Ruggiero

**Affiliations:** ^1^Department of Neuroscience and Mental Health, Foundation IRCCS Ca' Granda Ospedale Maggiore Policlinico, Milan, Italy; ^2^“Aldo Ravelli” Center for Neurotechnology and Experimental Brain Therapeutics, Department of Health Sciences, University of Milan, Milan, Italy

**Keywords:** Parkinson's disease, COVID-19, pandemic restrictions, non-motor symptoms, anxiety, depression, sleep disturbances, quality of life

## Abstract

This review discussed the effects of the impact of the Coronavirus Disease 2019 (COVID-19) pandemic on the psychological wellbeing of people with Parkinson's disease (PD) focusing specifically on depressive symptoms, anxiety levels, sleep, and quality of life (QoL). Together with motor symptoms, psychological symptoms are common and disabling conditions in the clinical course of PD becoming a relevant topic as a result of the lockdown measure due to alter their everyday life. We searched on PubMed online electronic databases for English articles published between January 2020 and 31 December 2021. Twenty-eight relevant studies were found and included in the review. Heterogeneous data emerged from the topics analyzed. Overall, data from depression studies showed significant depressive symptoms if the patient was analyzed longitudinally or vs. a control group consisting in healthy subjects, while these differences become minimal when the control group is a family member. Differently, in most of the studies reviewed there is no evidence of a statistically significant impact on anxiety disorders, nor on the quality of sleep. Conversely, PD patients showed a statistically significant negative impact of QoL compared with control groups or other neurological conditions. Although these findings must be interpreted carefully in the light of the studies' limitations, both in methodology and design, collectively our review showed that COVID-19 pandemic has had negative effects on the mental health of people with PD, due to disruption of healthcare services, loss of usual activities and supports and reduction in physical activity.

## Introduction

In December 2019, the first cases of a disease called Severe Acute Respiratory Syndrome Coronavirus 2 (SARS-CoV-2) were identified in Wuhan, in the province of Hubei, China (Del Prete et al., 2021). Available data suggest that the outcome of the new Coronavirus Disease 2019 (COVID-19) can be worse in elderly people and patients vulnerable due to pre-existing conditions (Hall and Church, 2020).

Faced with the spread of the virus and the growing pressure on healthcare facilities and workers, hospitals also had to limit patients' access, a decision that greatly impacted routine diagnosis and treatment of chronic diseases, including Parkinson's disease (PD) (Guo et al., 2020), a neurodegenerative disease characterized by movement disorders, cognitive impairment, vulnerability, and comorbidities.

Healthcare services provided to patients with PD were swiftly adapted to the restrictive measures in place (Brown et al., 2020). In fact, to ensure continuity in healthcare, alternatives to the classic in-person visit have been adopted, including telemedicine (Bloem et al., 2020) and remote advanced therapeutic management (Fasano et al., 2020; Miocinovic et al., 2020), taking into account differences in accessibility and usability for some patients (Garg and Dhamija, 2020).

Besides the motor symptoms, neuropsychiatric disorders such as anxiety, depression, apathy, and sleep disturbances, are common and highly disabling in the clinical development of PD (Weintraub and Burn, 2011) becoming even more relevant as a result of the lockdown measure. PD patients, as well as other at-risk groups, were forced to alter their everyday life in a way that deeply affected their social interactions, routines, and physical training, which normally allow PD patients to reduce the stress associated with their condition and to optimally cope with the disease (Haahr et al., 2011; Corti et al., 2018). Normal dopaminergic functioning is essential to a flexible adaptation to new circumstances (Macht et al., 2007; Robbins and Cools, 2014), which means that PD patients may be more likely to suffer from the negative psychological and psychosocial effects of self-isolation and of other measure in place to contain the pandemic, thus negatively affecting the disease burden (Helmich and Bloem, 2020; Papa et al., 2020).

The aim of the present review was, therefore, to identify and narratively summarize available studies, conducted in different countries, investigating the potential impact of the COVID-19 pandemic and subsequent social restrictions on non-motor symptoms (NMS) in patients with PD, focusing specifically on depressive symptoms, anxiety levels, sleep, and quality of life (QoL).

## Methods

To identify available studies, a search was carried out through the PubMed/Medline online database on articles published between January 2020 and the end of December 2021. The choice of this time interval was dictated by the World Health Organization who had declared the international SARS-CoV-2 outbreak a Public Health Emergency of International Concern on 30 January 2020.

The designed search strategy resorted to the use of MeSH terms and keywords to search the database for the disease in combination with the pandemic context and NMS [disease (e.g., Parkinson's Disease) AND context (e.g., COVID-19)] AND [NMS (e.g., anxiety)].

**Figure 1:**
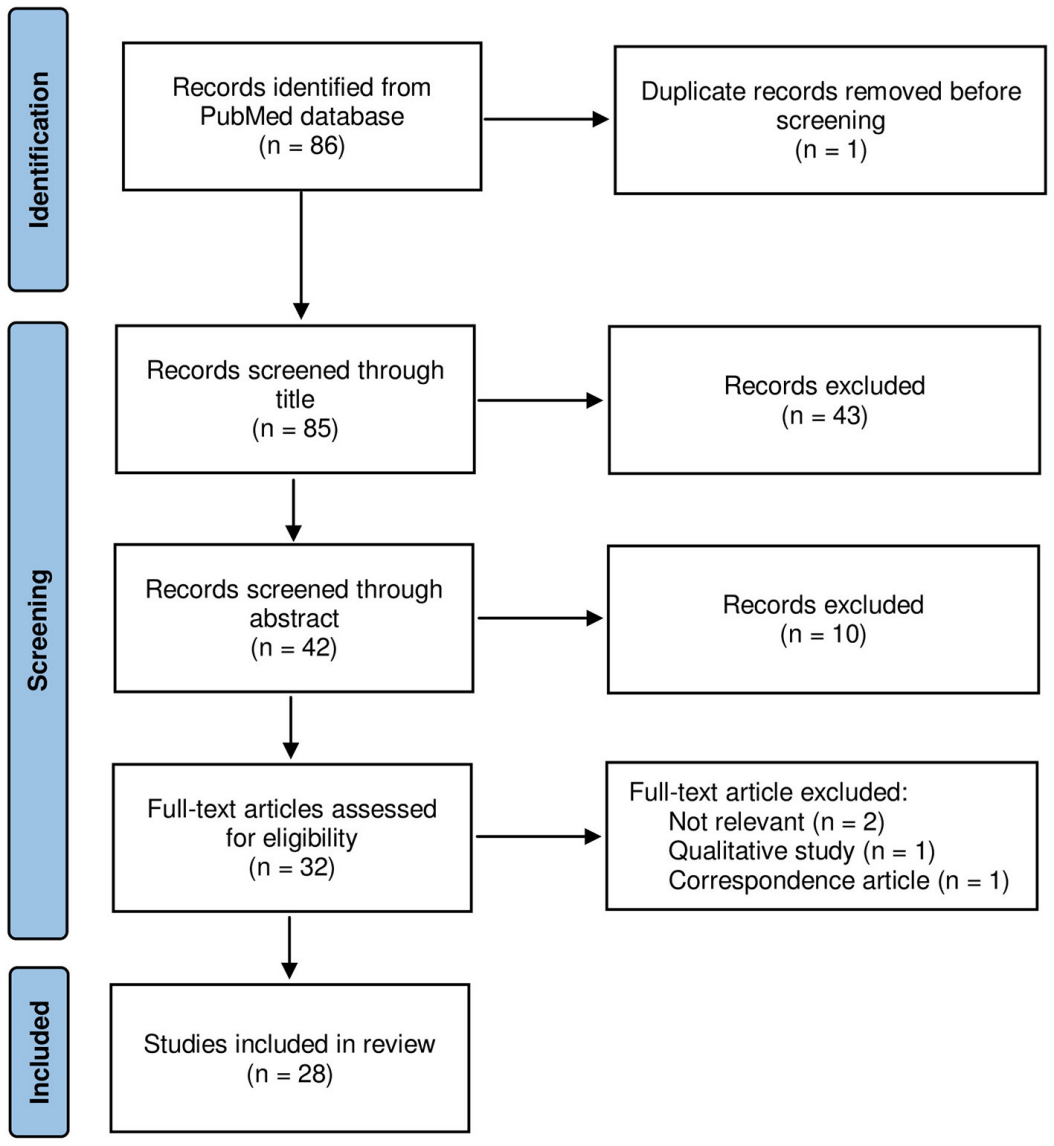
Flowchart of the studies' selection process.

Two independent reviewers and qualified researchers in clinical psychology screened records of search outputs for pertinence to the topic and English language only. A flow chart of the systematic literature search is reported in Figure 1.

Experimental studies were included if they addressed the impact of the COVID-19 pandemic restrictions on depressive symptoms, anxiety, sleep, and QoL in patients with PD. No restrictions were applied as to sex, age and ethnicity of subjects, disease duration, and disease severity. Reviews, qualitative studies, correspondences, single case reports and studies written in languages other than English were all excluded. The articles passed a first screening phase, checking titles and abstracts, and a second screening phase, analyzing the full-text if they met the above criteria.

Since some of the studies investigated several NMS at once, we extrapolated data on each one of them. The present review is structured into sub-sections dedicated to individual topics (depression, anxiety, sleep, and QoL) and therefore the same study might figure in more than one sub-section. Finally, within each sub-section, we organized the results according to the experimental design of symptoms evaluation: assessment of patients before and after lockdown, comparisons between patients and control groups, and individual assessments conducted only in the PD patient group.

## Results

After checking for duplications and compliance to selection criteria, 28 studies focusing on the impact of COVID-19 restrictions on depressive symptoms, anxiety levels, sleep, and QoL were included in the review process (out of the 86 initially scrutinized articles). Figure 1 illustrates the search and selection process. The selected studies were conducted in different countries, where restrictions started at different times and with different degrees of strictness (e.g., recommended social distancing, compulsory lockdown). Most studies evaluated PD patients' symptoms during or immediately after the first wave of COVID-19 infections, resorting to different validated scales or questionnaires specifically designed to assess subjective perception of change in NMS. Only two studies included in this work collected data during 2021, following successive waves of COVID-19. Results are narratively summarized below, in sub-sections focusing on depression, anxiety, sleep, and QoL. An overview of the data extracted from each study is detailed in Supplementary Table 1.

### Depression

#### Longitudinal monitoring

A study by HØrmann Thomsen et al. (2021) assessed depression between August–November 2018 (baseline) and April–June 2020 (follow-up) in two different samples, using the Beck Depression Inventory-II (BDI-II) for Danish patients and the Patient Reported Outcome in Parkinson's Disease (PRO-PD) for Swedish patients. The results showed no statistically significant differences between the two assessments for either cohort; Swedish patients reported lower scores for depression at baseline, whereas in the Danish cohort the scores were almost identical between the two evaluations. Only one parameter indicated a worsening of mood: by analyzing the scores obtained on the Parkinson's Disease Questionnaire-8 (PDQ-8), the authors found a worsening at T1 in the “felt depressed” item in both cohorts. However, considering that data at T0 were collected 2 years before the pandemic, it is indeed possible that other variables might have come into play.

To assess depression symptoms, El Otmani et al. (2021) evaluated 50 PD patients at the start of lockdown in Morocco (16 March 2020) and again after 6 weeks of home confinement, by online submission of the Moroccan version of Hospital Anxiety and Depression Scale (HADS). Their results showed no differences either in the mean scores of depression subscale of HADS (HADS-D) or in the total number of depressed patients. More specifically, at follow-up they observed that 5 patients improved their HADS-D score while 12 showed a clinical worsening of depression, a change in both cases not correlated to disease severity. Among the 12 worsening patients, 8 of them were comparatively young ( ≤ 60 years old) and 3 had already undergone Deep Brain Stimulation (DBS) treatment; thus, as reported by telephone interview at follow-up, their worsening could well have been a consequence of the restrictions in place, which caused a reduction of physical activity and changes in daily routine.

Finally, Falla et al. (2021) evaluated 14 PD patients [disease severity ≤ III according to the modified Hoehn and Yahr scale (H&Y)] by administering the Geriatric Depression Scale (GDS) *via* telemedicine visits, and compared the data acquired during the last week of lockdown in Italy (24 April−1 May 2020) with those obtained during an evaluation conducted in February 2020, shortly before the lockdown. Results showed that patients did not exhibit depression, and no statistically significant difference between the two assessments was found. However, by analyzing individual scores, 4 out of the 5 patients who showed a clinically significant score for depression at baseline were found to still show the same score at follow-up. These data are consistent with existing studies pointing to a high incidence of affective disorders, including depression, in PD patients. No association was found between the difference from baseline to follow-up of GDS score and disease duration.

#### Case-control studies

To investigate the impact of the COVID-19 pandemic on depressive symptoms, Shalash et al. (2020) administered the Depression, Anxiety and Stress Scale-21 (DASS-21) by telephone to 38 PD patients and 20 controls in Egypt; the control group included volunteers and relatives of patients suffering from other medical conditions. They found that more PD patients (60.5%) than controls (30%) showed significantly mood deflection. Moreover, DASS-21 score for depression showed a positive correlation with pre-lockdown motor severity–off and with pre-lockdown Beck Depression Inventory (BDI), and a negative correlation with pre-lockdown Mini Mental State Examination. However, the study does not clearly state when the pre-lockdown evaluation was performed.

A study by Xia et al. (2020), conducted on a cohort of 119 Chinese PD patients and 169 healthy subjects, retrospectively investigated the levels of depression experienced between February and April 2020 by administering the HADS-D *via* an online survey. According to the results, patients showed significantly higher HADS-D scores when compared to controls. Additionally, patients affected by sleep disorders were more likely to show a worsening in depression levels. Furthermore, when comparing data by gender, it emerged that more females (*n* = 20, 34.5%) than males (*n* = 7, 11.5%) manifested clinical levels of depression (*p* = 0.003).

In order to investigate the effect of increased stress levels caused by the pandemic on patients' mood, the authors of the study by Oppo et al. (2020) administered the HADS to 32 PD patients and to their caregivers through a structured telephone interview conducted over the course of the last 10 days of the first lockdown in Italy (may 2020). The authors found no statistically significant differences between the two groups in the mean HADS-D score. However, results showed that patients who subjectively experienced worsening stress levels due to isolation also showed significantly worse mood/cognition scores on the Non-Motor Symptom Scale (NMSS). This outcome may be explained by the fact that the items belonging to the mood/cognition domain cannot be unbundled, therefore it is impossible to clearly establish whether the worse score is due to the cognition or to the mood component.

In line with these findings, Suzuki et al. (2021) performed retrospective evaluations between June and December 2020 through an online survey, asking 100 Japanese patients and their caregivers/spouses to answer the questions by focusing only on their conditions after mid-April 2020, when the health emergency was declared. HADS was administered to both patients and caregivers to assess depression, while the Patient Global Impression of Change (PGIC) scale was applied to patients with PD to assess the impact of the pandemic on motor symptoms and mood. In addition, caregivers/spouses of PD patients were asked about the changes in the patients' symptoms they observed. The HADS score results showed no statistically significant differences between the percentage of patients (*n* = 20, 20%) and caregivers (*n* = 17, 17%) who suffered from clinical depression during the COVID-19 pandemic, while PGIC results revealed that, whereas 36% of patients (*n* = 36) reported mild to moderate mood worsening, 56% (*n* = 56) reported no change, while only 8% (*n* = 8) reported mild to moderate improvement. Additionally, a high rate of agreement was observed between patients' accounts and caregivers' reports of symptom change.

Kitani-Morii et al. (2021) invited 88 people to participate in a survey. The response rate was 80% (*n* = 71). They assessed depressive symptoms in 38 PD patients and 31 of their relatives through the Japanese version of the 9-item Patient Health Questionnaire, sent to the subjects by email (from 22 April to 15 May 2020). The results highlighted that more patients (*n* = 15, 39%) than controls (*n* = 2, 6%) showed moderate to severe depressive symptoms (*p* = 0.002), and that more female patients (*n* = 7, 50%) than females in the control group (*n* = 1, 3.8%) had depression (*p* = 0.001). Additionally, duration and severity of disease did not emerge as risk factors for clinical depression, while the patients' perceived severity of motor symptoms did.

A telephone interview was conducted on 45 patients and 43 healthy controls after the end of the lockdown in Turkey (15–20 June 2020) by Balci et al. (2021), asking the participants to refer to the period 11 March−1 June 2020. Depressive symptoms were evaluated by administering the HADS in relation to physical activity measured with the Physical Activity Scale for the Elderly. Their results showed that, although a worsening of motor and NMS was reported by 31 patients (68.9%), no statistically relevant mood differences or deviations in the HADS-D score were found, not in patients nor in healthy subjects.

Lastly, Montanaro et al. (2022) enrolled 100 PD patients undergoing different therapies [DBS, levodopa/carbidopa intestinal gel infusion (LCIG), standard medical treatment (SMT)] and 60 caregivers. The authors administered HADS-D *via* telephone interviews during the lockdown in Italy (April–May 2020), to both patients and caregivers. Patients were evaluated at T0 (April–May 2020) and at T1 (June–August 2020), while caregivers were only evaluated at T0. Results showed that more patients (35%) than caregivers (21.7%) experienced depression, and a significantly higher mean HADS-D score was observed in patients compared to caregivers during the 2 months of lockdown in Italy (T0). Comparing the two patients' assessments, 35% of them showed depressive symptoms at T0, with a mild, medium, or severe intensity, and a similar trend was observed at T1 (34.1%), suggesting no change happened in patients' depression levels. Moreover, no statistically significant difference in depressive symptoms was observed both at T0 and T1 between patients treated with DBS, LCIG, and SMT, and no correlation was found between HADS-D score and disease duration. However, the absence of a pre-pandemic assessment of both groups and also of a post-lockdown assessment of caregivers prevents further comparisons, therefore it is impossible to establish whether the higher incidence of depressive symptoms in patients is actually related to the restrictions in place or if it is due to pathophysiologic predisposition or progression of PD.

#### Cross-sectional studies

Song et al. (2020) evaluated the change in NMS by asking the patients a single question: whether they felt “subjectively” depressed or not. In the study were enrolled 100 patients with PD (H&Y stage ≤ III) who were able to walk independently and who visited their clinic a first time between December 2019 and January 2020 (baseline) and a second time in May 2020 (follow-up during the COVID-19 crisis). Results showed a low impact of the pandemic on depressive symptoms: only 5% of the patients reported feeling subjectively depressed.

A study by Guo et al. (2020) investigated the insurgence or worsening of depressive symptoms on February-March 2020 with a two-question survey, administered to 113 patients with PD (H&Y stage I-IV). The response rate was 95.6% (*n* = 108). Among the 86 (79.6%) patients who reported new or exacerbated symptoms, 54 (50%) indicated “feeling depressed” as the main trait; it was also the most reported symptom. However, the authors did not explore the possibility of a correlation between the subjective perception of depression and the stage of the disease, and with no pre-COVID-19 data it is impossible to determine whether the worsening is due to the pandemic or rather to the progression of the disease.

Similar findings emerged from the study of Janiri et al. (2020), who conducted a telephone survey in April 2020 on 134 PD patients, 101 of which (75.4%) already suffering from long lasting psychiatric symptoms. All participants answered the interview questions. Results showed that a worsening of psychiatric clinical condition during COVID-19 outbreak was reported by 23 (22.8%) of the 101 patients, while the others reported no change. Almost all of the patients (*n* = 19, 82.6%) with worsening symptoms reported a worsening in depression. In line with these findings, a case-control survey of COVID-19 and other clinical features in PD patients living in Tuscany was conducted by Del Prete et al. (2021) on 740 subjects. All subjects were telephonically interviewed from 10 April to 4 May 2020 by a neurologist specialized in movement disorders; patients were asked about COVID-19 positivity, comorbidities, anti-Parkinson's therapy, mood, and other clinical signs. Among the 733 (99%) non-COVID-19 patients, the majority (*n* = 549, 74.9%) did not notice any pandemic-related mood worsening. However, this outcome was gathered through a single question. Additionally, DASS-21 was performed on 120 non-COVID-19 patients but no specific data were available for the depression subscale.

Fabbri et al. (2021) investigated perceived changes in depressive symptoms by resorting to a survey designed by the France Parkinson association to specifically target the French community of PD patients, which was administered online or as part of classic routine consultation or outpatients' telemedicine visits performed by a hospital-based Parkinson Expert Center. All patients were evaluated from 16 March to 16 May 2020, assessing changes occurred between mid-March to mid-April. The authors used the same standardized questionnaire, assessing both motor symptoms and NMS (including depression) by administering the Patient's Global Impression-Improvement scales (PGI-I) and asking patients to report new/worsening symptoms. Results showed that, out of the 2,653 responses analyzed, 1,085 (40.9%) patients reported a change in symptoms and only 3.4% (*n* = 90) of all patients reported a worsening of depressive symptoms. However, according to the PGI-I, worsening of the psychic state (including depression and anxiety) was among the most reported symptoms (*n* = 1,211, 46.3%), in addition to general motor symptoms (*n* = 1,451, 55.8%) and pain (*n* = 1,336, 51.5%). Nevertheless, as only few patients reported a change in their depressive symptoms, the result of this scale could have been influenced more by a worsening of anxiety.

A study conducted in India by Saluja et al. (2021) assessed the presence of depression symptoms: over the course of a telephone interview (June–September 2020), the authors administered the NMSS to 64 patients and asked their caregivers to report the changes they had perceived in the patients' symptoms. The authors found agreement among patients and caregivers in reporting an increase in sadness and low mood. Mood disturbances were the most common NMS to worsen: more specifically, 26 patients reported a worsening of symptoms and 42.3% of these reported an increase in mood deflection. The impact of lockdown on NMS might have been critical in patients with PD as dopamine depletion can already lead to cognitive inflexibility, inability to adapt, and reduced ability to cope with stress (Saluja et al., 2021).

Kumar et al. (2021) investigated the impact of the pandemic on depression symptoms by assessing sleep as an indirect measure of mood. The authors administered an online survey to 832 patients through a specifically tailored questionnaire, created and validated by them in order to assess changes in sleep parameters, together with mood. Analysis on all 832 responses showed that patients who experienced new or increased sleep disturbances following the lockdown were characterized by a higher prevalence of depressive symptoms (*n* = 112, 56.3%), compared to those with no sleep disorders (*n* = 192, 35.7%) and patients with pre-existing sleep disorders that did not exacerbate during the pandemic (*p* < 0.001). This study also suggests that there is indeed a relationship between depression and sleep disturbances.

In a study conducted in Canada (de Rus Jacquet et al., 2021), 417 PD patients from two cohorts (median H&Y stage II), 177 from Alberta and 240 from Québec, were enrolled by three associations: the Canadian Open Parkinson Network, the Quebec Parkinson Network, and the Calgary Parkinson Research Initiative. Participants were asked to fill an online survey, available from 20 May to 16 September 2020, about the impact of COVID-19 confinement (starting from mid-March 2020) on perceived physical and mental wellbeing, daily activities, disease management, and continuity of health care. Questionnaires were self-administered online or on the telephone by researchers. Changes in mood ranked third in the list of the main effects of confinement. Among patients who perceived a change in depression level (*n* = 56, 13.4%) the majority of them (*n* = 38, 67.86%) reported an increase in depression, while the others (*n* = 18, 32.14%) registered only day-to-day changes.

A study by Knapik et al. (2021) assessed depression levels in 30 PD patients (H&Y stage I–III, none of whom had previously received DBS treatment) 90 days after the onset of social isolation in Poland. From the administration of the HADS by telephone interview, they observed that 40% of patients did not report depression, that 33.33% of them had a borderline HADS-D score for the presence of that disorder, while the remaining 26.67% had clinical depression. In this study, the high levels of depression may be partially explained by the patients' perception of their physical fitness, but not by the physical activity they actually engaged in, while staying at home or when leaving home during the pandemic. The authors also divided the patients into two groups, “people living alone” and “people living with a spouse or family” (*n* = 6 vs. *n* = 24), and compared the mean HADS-D scores of the two subgroups. No statistically significant differences emerged and this could perhaps be due to the fact that the two groups had different sample sizes.

In a study by Dommershuijsen et al. (2021), 844 PD patients answered a questionnaire administered online and, in a few cases, on paper or by telephone, from 14 April 2020 to 25 February 2021, aimed to identify subgroup differences in the associations between COVID-19 stressors and mental health in PD patients. Specifically, when focusing on the relationship between pandemic-related stressors and depression, the authors found that higher care stressors (e.g., limited access to care, nursing, and medication), social stressors (e.g., reduced social contact, lack of social events, and tension/conflict at home), and stressors sum score were associated with higher BDI total score (BDI beta: 0.07, 95% CI: 0.04–0.10; BDI beta:0.06, 95% CI: 0.04–0.08; 95% CI: 0.02–0.05) and sub score. The authors also measured a greater impact of care stressors on depression levels in women rather than men, and on patients suffering from more severe PD symptoms.

Finally, Krzysztoń et al. (2022) made an online survey available between 23 December 2020 and 23 June 2021, asking several questions aimed at investigating the impact of the COVID-19 pandemic on PD patients. Forty-seven patients completed the survey and 9% (*n* = 4) of them reported subjective worsening of depressive symptoms. Additionally, a statistically significant correlation was found between the worsening of depressive symptoms and the feeling of being alone/isolated (*p* = 0.017).

### Anxiety

#### Longitudinal monitoring

A study conducted in Italy by Falla et al. (2021) highlighted that, in their sample (H&Y stage ≤ III), more PD patients reported anxiety at follow-up during lockdown (T1) compared to baseline performed shortly before the lockdown beginning (T0). Furthermore, the mean of the 12-item Parkinson Anxiety Scale (OR-PAS) total score was higher at T1 (16.9 ± 7.4) than at T0 (11.8 ± 8.4) and, more specifically, the avoidance subscale significantly increased at T1 (7.5 ± 1.7) compared to T0 (1.8 ± 2.2). Thus, anxiety levels measured at baseline were already above the cut-off, further worsening during the lockdown period. Because no association was found between the difference in OR-PAS score from baseline to follow-up and disease duration, this increase may be a consequence of the social isolation experienced during the lockdown in addition to the restrictions.

In line with this result, another study conducted on two different PD cohorts, one Swedish and one Danish (HØrmann Thomsen et al., 2021), showed a statistically significant worsening of anxiety between the baseline period (2018) and the first COVID-19 wave in both cohorts, although in the Danish cohort just one item extracted from the BDI-II scale was used as a measure of anxiety. However, qualitative data from narrative reports about the impact of COVID-19 on everyday life provided by Danish patients supported this finding. In particular, these descriptive texts suggest that the increase in anxiety may be associated with the hardship of rebuilding a daily routine after this challenging period. It is worth noting that, as this study compared data collected in 2018 with an assessment during the social isolation period, differences should be treated with caution since multiple events may have occurred in a 2-year timeframe, possibly impacting on anxiety levels measured during the social restrictions period. Besides, such an increase in the anxiety levels of patients over the course of time has not been found in other studies.

Contrary to the aforementioned results, the assessment of a sample of 50 PD patients in Morocco (El Otmani et al., 2021) showed that, after 6 weeks of home confinement, the anxiety subscale of the HADS (HADS-A) showed a substantial stability on anxiety levels. In fact, 32% of patients reported anxiety at the beginning of confinement, and the mean HADS-A score was 7.98; similarly, after 6 weeks of self-isolation, 30% reported anxiety and the mean HADS-A score was 8.24. Thus, the authors found no statistically significant differences for anxiety levels, either by comparing the patients' ratio or the mean HADS-A scores in the two assessments. Noteworthy, out of the 8 patients who showed a worsening in anxiety levels, 7 were under the age of 60 and 2 had undergone DBS treatment: the negative effect observed in younger patients and in patients treated with DBS may be explained by the impact of the governmental restrictions on daily routines witch limited their opportunities to exercise. The authors found that disease severity had no impact on the HADS-A score. Thus, despite the trend toward an increased anxiety symptomatology seen in previous studies, this study showed that confinement did not have a statistically significant impact on anxiety scores: the overall effect on anxiety seems null, although in this sample some patients improved and others worsened.

#### Case-control studies

Among the studies that produced statistically significant results, there is an online case-control survey which included the Persian version of Beck Anxiety Inventory-II (BAI-II), conducted online by Salari et al. (2020). In this study, based on a sample of 137 PD patients, 95 caregivers, and 442 healthy controls, the authors concluded that the mean BAI-II total score was consistently higher in PD patients than in both controls and caregivers (*p* < 0.001). Similarly, moderate to severe anxiety levels were more frequently observed in PD patients than in controls and caregivers. PD disease duration was also considered in this study, but no statistically significant correlation was found between this parameter and the severity of anxiety levels. The authors argue that this outcome could be due to worsening of pre-existing anxiety, uncertainty of treatment during isolation, and increased vulnerability to COVID-19 due to a chronic condition.

A study by Shalash et al. (2020), collected data from 38 PD patients and 20 age- and sex-matched controls (volunteers and patients' relatives) by administering on the telephone a version of the DASS-21 and asking a few additional questions to investigate PD patients' perceptions of the impact of the first wave of the COVID-19 pandemic. The authors found that PD patients showed significantly higher mean DASS-21 anxiety score and prevalence of anxiety (*n* = 23, 60.5%) compared to controls (*n* = 5, 25%). In addition, they found a positive correlation between anxiety and pre-lockdown off-motor severity. Instead, from the subjective report, it appeared that 20 patients (52.6%) reported anxiety/stress caused by COVID-19. Although the causes for subjective anxiety and stress cannot be disaggregated, the paper concluded that anxiety is frequently aggravated by restrictions.

However, it is worth noting that neither of the two aforementioned studies (Salari et al., 2020; Shalash et al., 2020) mention the data collection period.

Conversely, Oppo et al. (2020) failed to find a statistically significant difference between PD patients' and caregivers' anxiety levels, also in relation to disease duration. Moreover, by dividing the sample into two groups, active patients—who continued to engage themselves in physical activity—and inactive patients, and comparing them, the study concluded that the first group had significantly lower anxiety levels than the second one. The results indicated that physical activity could exert an influence on anxiety levels: in fact, a statistically significant inverse relationship between anxiety and physical exercise was observed and, furthermore, anxiety was the only statistically significant determinant for the subjective assessment of worsening stress caused by lockdown in PD patients, measured through a verbal rating scale.

In a quantitative study, Xia et al. (2020) studied the impact of the COVID-19 pandemic on several dimensions, including anxiety, through the HADS. Compared to healthy controls (*n* = 29, 17.2%), PD patients (*n* = 25, 21%) did not have a significantly higher prevalence of anxiety (*p* = 0.410). Moreover, patients with sleep disorders showed a higher anxiety level than those who did not experience sleep disturbances, indicating that anxiety has a powerful effect on sleep quality. Furthermore, results showed that females (*n* = 19, 32.8%) reported a significantly higher incidence of anxiety levels compared to males (*n* = 6, 0.1%, *p* = 0.002), suggesting that female patients are more likely to experience anxiety.

Kitani-Morii et al. (2021) compared anxiety levels in 39 PD patients and 32 family members through the administration of the 7-item Generalized Anxiety Disorder by email without finding differences. Participants were also asked on the telephone if they had subjectively experienced a worsening in anxiety levels and the response rate was 100%. Although PD patients (*n* = 19, 48%) were significantly more likely to suffer from clinical anxiety than controls (*n* = 11, 34%) results revealed that, among both patients and controls, 36.6% (*n* = 26) reported subjective worsening of anxiety and, surprisingly, the percentage was higher in controls (*n* = 14, 43.7%) than in PD patients (*n* = 12, 30.7%), but still not in the range of statistical significance (*p* = 0.25). Overall, results suggest that PD patients, especially those potentially suffering from pre-existing psychological issues, may be more likely to develop clinical anxiety in response to social distress; although, due to the lack of data on the patients' neuropsychiatric status prior to the COVID-19 pandemic, the study fails to demonstrate that the COVID-19 pandemic worsened the neuropsychiatric status of patients with PD.

In a study by Suzuki et al. (2021) that investigated the determinants of QoL on 100 PD and their caregivers, the HADS was administered to assess anxiety. The prevalence in both groups was similar: in fact, anxiety was observed in 6% (*n* = 6) of both PD patients and caregivers, while other factors, such as disease severity, impacted negatively on patients with PD and were overall related to the worsening of their QoL. These findings may also indicate that, during the lockdown, the caregiver's daily burden intensified, and this may have contributed to the rise of anxiety levels not only in patients, but also in caregivers.

Balci et al. (2021) examined the severity of anxiety in PD patients compared with healthy individuals under lockdown conditions. Contrary to expectations, neither patients nor controls showed an increase in anxiety levels; in fact, the 45 patients and 43 healthy subjects showed similar median HADS-A scores and, more importantly, there was no difference between the two groups.

Lastly, Montanaro et al. (2022) assessed anxiety levels in 100 advanced PD patients who were experiencing severe symptoms and motor complications and in 60 caregivers by administering on the telephone the HADS-A between April 2020 and May 2020, while social restrictions were in place (T0). Subsequently, only PD patients were re-evaluated after the lockdown ending, between June 2020 and August 2020 (T1). The authors found that both groups showed a similar prevalence of anxiety symptoms (39% PD patients vs. 40% caregivers) and no statistically significant difference emerged on the mean HADS-A score at T0. Comparing the two patient assessments, fewer subjects from this sample showed anxiety at T1 after lockdown (30.6%) than at T0 (39%) (*p* = 0.023). Furthermore, among the patients enrolled in this study, there were subjects treated with DBS, LCIG, or SMT, so potential differences in anxiety symptoms between patients undergoing different treatment were also evaluated; a statistically significant correlation emerged between anxiety and different therapies: HADS-A scores were higher in SMT and LCIG than in DBS patients at T0, while at T1 a significance was only found for LCIG. This study highlighted how different treatments have different psychological effects. In fact, patients treated with DBS showed lower levels of anxiety than the other groups, probably due to the fact that DBS devices can be handled more independently. This study provides a snapshot of anxiety status in the middle of restrictions, however it only allows for comparison between groups at T0 and not after lockdown at T1, as caregivers were not re-assessed, nor are there any pre-pandemic measures to interpret the data collected during lockdown.

#### Cross-sectional studies

van der Heide et al. (2020) assessed whether the COVID-19 pandemic was associated with increased psychological distress, and if and how these changes may prove useful in identifying predictors of increased psychological distress in PD patients during health emergencies. Resorting to the subscale of the Parkinson Anxiety Scale (PAS), the researchers questioned 358 patients through an online survey (response rate was 71.9%), who reported higher levels of stress and anxiety during the pandemic. Moreover, in a retrospective comparison, they found a statistically significant and strong correlation between the Perceived Stress Scale score, the PAS episodic anxiety subscale score (both administered during lockdown), and the State-Trait Anxiety Inventory (STAI) score collected before the COVID-19 pandemic. These results indicate that the presence of anxiety before the pandemic led to a higher perceived stress and a greater manifestation of anxiety symptoms during the lockdown. Additionally, the authors of this study reasoned with the relation between psychological distress and symptom severity, arguing that motor symptoms are particularly sensitive to stress, as stated by previous work (Boman, 1971; Zach et al., 2015), as well as to the reduction of physical activity.

In a study by de Rus Jacquet et al. (2021), based on an online survey, the authors observed that 125 (29.7%) of a sample of 417 Canadian PD patients (median H&R stage II) reported subjective changes in anxiety levels after lockdown. Among them, 94 (75.2%) reported an increase in anxiety and 29 (23.2%) reported daily changes. Only two patients reported a reduction of anxiety levels.

Knapik et al. (2021) evaluated a group of Polish patients (H&Y stage I-III, not subjected to DBS) after about 3 months of social restriction: half of them exhibited no anxiety levels to HADS-A. Among the remaining patients, 30% had a borderline score and 20% showed anxiety which, according to the study, was only partially explained by patients' self-assessment of their physical fitness, but not by the actual physical activity they engaged in. Furthermore, the authors failed to find statistically significant differences in the mean HADS-A score between patients living alone and patients living with relatives.

A questionnaire-based study by Kumar et al. (2021), focused on sleep disturbance, found a significantly higher prevalence of anxiety symptomatology among patients who experienced an onset or exacerbation of sleep disturbances during confinement (*n* = 119, 60%) compared with patients who did not (*n* = 210, 39.1%) or patients with pre-existing sleep disorders that did not worsen during the pandemic (*p* < 0.001). Anxiety is known to induce hyperarousal that is pathophysiologically associated with insomnia. However, lacking a measure of anxiety levels at baseline, the study cannot determine whether anxiety was a predisposing factor for the development of sleep issues during lockdown or, vice-versa, if sleep issues arisen during lockdown triggered the onset of anxiety.

Dommershuijsen et al. (2021) administered a questionnaire to investigate potential associations between COVID-19 stressors and anxiety levels measured through the STAI. They found that higher care stressors (STAI beta: 0.06, 95% CI: 0.04–0.09), social stressors (STAI beta: 0.06, 95% CI: 0.04–0.08) and stressors sum score (95% CI: 0.02–0.05) were associated with higher STAI total score and subscore. They also highlighted that social stressors had a greater impact on anxiety levels in patients with a higher level of education, while care stressors had a greater impact on anxiety in women and on patients with a disease duration longer than 5 years and with more severe PD symptoms.

Although anxiety was not the focus of their study, Del Prete et al. (2021) found that 74.6% (*n* = 547) of a sample of 733 PD patients did not experience a subjective worsening of anxiety during lockdown. Furthermore, as mentioned in a previous sub-section (see Cross-Sectional Studies), the study reported a mean DASS-21 total score that cannot be disaggregated into subscales, therefore not informative about patients' anxiety status. For this reason, it is not possible to make a comparison between the subjective experience of the patients and the results of the standardized scale, nor was a baseline value of the DASS-21 recorded to verify the presence of a change in anxiety levels.

With the purpose of evaluating changes in motor and NMS occurring after the first month of the 2020 lockdown (mild-March to mid-April 2020) as well as its psychosocial impact, Fabbri et al. (2021) administered validated scales (PGI-I scale, PDQ-8) and a self-report questionnaire both online or during a routine follow-up consultation by a movement disorder specialist. Two thousand six hundred fifty-three patients were included in the analysis and, although the majority of them reported no subjective worsening of PD symptoms (*n* = 1,568, 59.1%), 6.1% (*n* = 162) reported a worsening of anxiety symptoms, with anxiety being the fourth most frequently worsened symptom and the first non-motor one. Unsurprisingly, the aggravation of psychic state alteration was probably related to several self-reinforcing factors, such as the concomitant aggravation of motor symptoms, which are particularly sensitive to stress and may cause an anxiety/depression aggravation, and the diffuse interruption of physiotherapy and of any outdoor physical activities.

In another study (Krzysztoń et al., 2022) conducted in Poland through an online survey, 38% (*n* = 18) of the 47 PD patients who agreed to participate reported feeling anxious since the onset of the pandemic. Particularly, it was found that patients who exhibited the highest levels of anxiety were those with shorter disease duration, who were not involved in any patients' associations, and who were more frequently looking for information about potential PD/COVID-19 interactions on the Internet. Additionally, reduced social contacts and feelings of isolation had a significant impact on anxiety (*p* = 0.035; *p* = 0.007). These findings could be explained by a worsening of existing anxiety, insecurity associated with taking medication during isolation, and an increased perceived risk of contracting SARS-CoV-2 due to age and chronic condition.

### Sleep

#### Longitudinal monitoring

An Italian study by Falla et al. (2021) investigated potential changes in sleep patterns using the Movement Disorders Society-Unified Parkinson Disease Rating Scale (MDS-UPDRS). Results showed a statistically significant increase in score during lockdown compared to before the onset of the pandemic (February 2020). In detail, 5 out of 14 patients reported worsening sleep issues, and 8 of them experienced daytime sleepiness. However, as discussed by the authors themselves, it is not possible to estimate whether the worsening of sleep quality was due to lockdown or to other disease-related factors, as no data were collected from a control group, possibly formed by healthy subjects.

Different results were found in the study by HØrmann Thomsen et al. (2021), who assessed sleep using the PRO-PD in a cohort of Swedish patients and also using a single item from the Parkinson's Disease Questionnaire-39 (PDQ-39) in a cohort of Danish patients. Results showed that a statistically significant improvement in the second evaluation was only found for the Swedish cohort. Indeed, analyzing individual scores, the authors found that almost all patients reported fewer sleep problems during social restriction, despite the ongoing of the health emergency: this may be explained by a change in routine and a decrease in perceived pressure in their daily-life (Frazier, 2000). Nevertheless, it is difficult to ascertain if the improvement in sleep quality was real because the baseline measures are too far behind and multiple factors may have affected the results.

#### Case-control studies

A study by Xia et al. (2020) administered the Pittsburgh Sleep Quality Index (PSQI) self-report questionnaire online to 119 PD patients and 169 healthy controls in China. Results showed that PD patients, compared with healthy subjects, reported significantly worse mean PSQI scores and a higher prevalence of sleep disturbance (PSQI > 5; *n* = 82, 68.9% PD patients vs. *n* = 75, 44.4% healthy controls, *p* < 0.001). More specifically, PD patients reported a statistically significant worsening in several sleep patterns: quality, sleep duration, sleep disturbance, and daytime dysfunction (*p* < 0.001). No statistically significant differences emerged for sleep latency, sleep efficiency, and use of sleeping medication. Moreover, analyses showed that anxiety and exacerbation of PD symptoms were possible risk factors for developing sleep problems and, furthermore, it was observed that female compared to male patients showed significantly worse total PSQI scores (*p* = 0.009), worse sleep quality and duration, and more daytime sleepiness, resulting in poorer sleep. In line with the literature, the results of the study highlighted the presence of greater sleep disturbances in PD patients compared to healthy controls. The absence of clinical data on patients, such as duration of illness or severity of symptoms, does not allow for the investigation of the impact on sleep in individual patients.

A study by Kitani-Morii et al. (2021) investigated insomnia in patients and in a control group consisting of family members. They administered the 7-item Insomnia Severity Index paper-based questionnaire by email, and also asked participants if they had experienced a subjective worsening of insomnia. No differences emerged between patients and controls, on either the standardized scale (*n* = 13, 33% vs. *n* = 7, 21%, *p* = 0.286) or the subjective report (*n* = 5, 12.8% vs. *n* = 3, 9.3%, *p* = 0.64). However, from a qualitative observation, patients presented moderate to severe insomnia symptoms more frequently than their family members.

#### Cross-sectional studies

Guo et al. (2020) measured the presence and the worsening of sleep disorders as a factor that may have negatively impacted on patients' QoL during the COVID-19 pandemic. The results showed that, out of 108 patients, 43 (39.8%) reported new or worsening sleep disturbance.

Following the COVID-19 outbreak, Janiri et al. (2020) used telephone surveys to assess subjective sleep disturbances, including insomnia and rapid eye movement sleep behavior disorder (RBD), in 134 PD patients. Among a total of 23 (22.8%) patients who reported the worsening of symptoms during lockdown, 12 of them (52.2%) reported worsened insomnia and 5 (21.7%) reported worsened RBD. Insomnia was the second most widespread symptom listed by patients.

In their study, Del Prete et al. (2021) administered an *ad hoc* questionnaire by telephone to investigate new and worsening PD symptoms, including insomnia, following the beginning of the 2020 lockdown in Italy. Most patients (*n* = 567, 77.4%) did not report new or worsening symptoms of insomnia as a consequence of COVID-19 restrictions.

Fabbri et al. (2021) asked patients to subjectively report the three most worrying new or worsened symptoms experienced since the start of the lockdown. Only 4.1% (*n* = 109) of patients, out of a cohort of 2,653, mentioned a worsening of sleep disturbances.

Song et al. (2020) investigated sleep quality as an indirect measure of the impact of limited physical activity due to pandemic. They asked patients if they felt a worsening of their Parkinsonian symptoms after the onset of the COVID-19 pandemic and, if so, to choose which motor and NMS had worsened the most; among these, sleep was also listed. Results showed that only 5 (5%) out of 100 patients reported reduced sleep hours.

In order to quantify the changes in sleep, Templeton et al. (2021) administered a self-report, *ad hoc* survey to 28 PD patients during or after the lockdown, in an unspecified timeframe. Patients were asked to indicate the perceived change in various motor and NMS caused by restrictions. The results showed that patients, on average, experienced a slight worsening of sleep, which was more frequent in females than males.

To retrospectively investigate the effects of social restrictions on sleep, Balci et al. (2021) studied a cohort of 45 Turkish patients through a telephone interview. They found that, among the older patients (≥65 years), 7 suffered from worsening sleep problems and 6 from daytime sleepiness, while only 2 of the younger patients (<65 years) were similarly affected; both of them reported worsening sleep problems but only one mentioned daytime sleepiness. Although this difference is not statistically significant, it may be associated with a longer disease duration and therefore a greater progression of NMS.

To investigate changes in sleep in PD patients after the beginning of lockdown, Suzuki et al. (2021) administered the PGIC scale through a survey at the end of the restrictions. They tested 100 patients, and also asked their caregivers and spouses to report any sleep changes they observed. The results showed that 41% (*n* = 41) of patients reported mild to moderate worsening of sleep and 48% (*n* = 48) reported no change. The authors found agreement between the patients' and caregivers' reports of sleep changes. However, the use of the PGIC scale to assess changes in sleep quality may not be sensitive enough to detect such changes.

Kumar et al. (2021) investigated the impact of lockdown on sleep parameters in a cohort of 823 Indian patients through an online questionnaire, *ad hoc* developed and validated by nine experts, all proficient in managing patients with PD following standard procedure. They found that 295 (35.4%) patients experienced sleep disturbances and 199 (23.9%) reported new issues or a worsening of existing symptoms in all sleep parameters investigated. Among the entire cohort, the most reported symptom was insomnia (*n* = 152, 51.5%), followed by restless legs syndrome (*n* = 73, 24.7%), RBD (*n* = 67, 22.7%), and sleep disordered breathing (*n* = 47, 15.9%). Moreover, by comparing patients with and without new or worsening sleep disturbances, the authors detected that sleep problems were significantly associated with duration of home confinement >60 days, duration of PD ≥7 years, inadequate family support during lockdown, worsening of most motor and NMS, poor QoL, and not engaging in exercise or hobbies during lockdown. In addition, low physical activity and screen time >3 h/day before and during home confinement were associated with new or worsening sleep disturbances (*p* = 0.01; *p* = 0.015).

To assess sleep problems, Saluja et al. (2021) administered a structured questionnaire (NMSS) after the end of the lockdown. Both patients and caregivers were interviewed, thus comparing the subjective perception of patients with that of their caregivers. Results showed that, among a total of 26 PD patients who reported worsening during lockdown, 34.6% of patients reported worsened insomnia and 7.7% hypersomnolence, and caregivers substantially agreed with their judgments.

de Rus Jacquet et al. (2021) investigated the impact of self-isolation on sleep through an online survey completed by 417 patients from two Canadian cohorts, one from Alberta and one from Québec. The majority of PD patients (*n* = 263, 63.37%) did not notice any change in sleep, whereas 117 of them (28.19%) reported worsened sleep quality. Only a small number of patients (*n* = 35, 8.43%) reported improved sleep quality during home confinement for COVID-19.

Finally, a study by Montanaro et al. (2022) evaluated the impact on sleep as a consequence of the pandemic. To reach this goal, the authors administered by telephone an *ad hoc* questionnaire in which patients were asked to indicate their degree of agreement or disagreement with the statement “I have more difficulty falling asleep.” The results showed that 54% of 100 patients reported no change in falling asleep.

### Quality of life

#### Longitudinal monitoring

Among the authors of longitudinal studies that reached statistically significant findings, Guo et al. (2020) conducted a detailed interview resorting to face-to-face assessments, telephone calls, and social media during and after COVID-19 prevention and control measures in China (1 February−31 March 2020). Through the PDQ-39, they found that PD patients (H&Y stage I–IV) showed a significantly worse QoL during the restrictions imposed to prevent the spreading of COVID-19, compared with the following stage of gradual slackening of the prevention and control measures. Although telemedicine has been predominantly used to provide care since the onset of the pandemic, patients referred that their main difficulty was not being able to get in touch with their physician. Reduced healthcare accessibility affected the QoL of chronic patients: it is the case of PD patients who, due to the lockdown, were unable to get doctors' advice or had to change their routine medication, resulting in poorer QoL.

Similar results emerged from a study by Song et al. (2020), in which the authors retrospectively collected baseline data from medical records dating from before the COVID-19 emergency (T0), and then assessed PD patients (H&Y stage ≤ III) in May 2020 (T1), during the restrictions issued by the Korean government. In order to assess patients' autonomy, the authors used the Schwab and England scale of Activities of Daily Living (SE-ADL): they found a significantly decreased mean SE-ADL score at T1 compared to the T0, indicating an increased difficulty in performing daily activities and, consequently, a worse QoL, although there was no increase in the Unified Parkinson's Disease Rating Scale part 3 score.

A discrepant result emerged from a study by HØrmann Thomsen et al. (2021). In the two groups examined (Swedish vs. Danish patients) a statistically significant improvement in QoL after the beginning of social restrictions was found at both PDQ-8 for the Swedish cohort and PDQ-39 for the Danish cohort. Data analysis suggests that there was an improvement in “ability to concentrate” and “difficulty getting around in public places,” and a parallel worsening in “close relationships” and “feeling depressed.” The improvement may be due to reduced social pressure, while the worsening of the last two items is consistent with the restrictions faced by the patients. More generally, this study, contrary to the previously mentioned studies and to expectations, highlights the possibility that some of the demands and impositions of pre-pandemic life may have inadvertently reduced QoL in PD patients.

Two longitudinal studies concluded for a non-impaired QoL in PD patients during the confinement. In the study performed by Falla et al. (2021), QoL was longitudinally assessed using structured questionnaires at baseline (in-person, February 2020) and at follow-up (*via* online video, during the lockdown in Italy, in spring 2020). The PDQ-39 summary index (PDQ-39-SI), filled out by 14 PD patients during the last week of the lockdown, showed no statistically significant difference compared to the pre-lockdown baseline. In addition, no statistically significant correlations were found between the difference in PDQ-39-SI score from baseline and follow-up and disease duration. The results could be explained by the fact that telemedicine evaluation appears to provide continuity of care, while also reducing the risk of infection in PD patients.

Likewise, in a sample of 12 PD patients no statistically significant findings on the impact of COVID-19 restrictions on QoL were found (Luis-Martínez et al., 2021): the study could not find statistically significant changes in PDQ-39, Activities of Daily Living, Instrumental Activities of Daily Living, and Parkinson's Disease Cognitive Functional Rating Scale completed 2 weeks before isolation (T0) and after 2 months of isolation (T1), on May 4, 2020. Authors supposed that the continuous contact with a multidisciplinary team during the lockdown contributed to the mitigation of negative effects on patients. Overall, the results of these two studies did not find an influence on QoL but, since both samples were small, they must be considered with reservations.

#### Case-control studies

Shalash et al. (2020) administered the PDQ-39 to 38 patients and 20 controls, but the paper does not state when they collected the data. In their sample, the total PDQ-39 score and most of the PDQ-39 domains, except for social support and communication, were significantly higher for PD patients compared with controls: therefore, PD patients had worse QoL. This result highlights that a worse QoL is associated with worse mental health status, an expected outcome consistent with the indirect impact of COVID-19.

Additionally, a study including 100 PD patients and 100 caregivers showed that, during the COVID-19 pandemic, PD patients suffered from a worsened QoL compared to controls (Suzuki et al., 2021). Authors registered the subjective point of view of PD patients and of their caregivers using the Japanese version of Short Form-8; subjects reported worse scores in half of the domains investigated by the aforementioned scale (physical function, role-physical, general health, vitality, and physical component summary). Additionally, the authors found that disease severity, disease duration, gait impairment, rigidity, anxiety, depression, and stress had a negative impact on QoL perception. Furthermore, researchers highlighted the relationship between patients' depression and stress and caregivers' negative mood. This finding demonstrates that the QoL of patients and their caregivers are closely related, and that the QoL of caregivers was also affected by the heavier burden experienced during the lockdown.

Another study (Sahin et al., 2021), collecting data during the lockdown in Turkey, which started in March 2020, compared the assessment of 98 PD patients and other patients suffering from various chronic neurological diseases, such as headache, multiple sclerosis, epilepsy, polyneuropathy, and cerebrovascular disease. The authors, through the Turkish version of the World Health Organization Quality of Life short form questionnaire, found lower scores in the social subscale that assesses the social relationships domain in PD patients compared to patients with other conditions, although data analysis did not show any statistically significant differences in the physical, mental, and environmental scores in both groups. This means that, for PD patients, lockdown had a greater impact on QoL in the social domain compared to other aspects of QoL: this was expected, presumably also because the PD patients' group was older and more vulnerable, therefore subjected to stricter isolation measures which severely reduced their social interactions. Conversely, the PD group showed the lowest distress level caused by the health emergency: this outcome suggests that isolation may have protected patients from COVID-19 infection (PD patients showed the lowest infection rates in the sample) and resulting post-traumatic symptoms.

#### Cross-sectional studies

Among the reviewed studies, the following works assessed the impact of COVID-19 self-isolation and social distancing on QoL in PD patients using the same tool: the PDQ-8 scale.

Oppo et al. (2020) studied the overall influence of home confinement on different PD symptoms and, therefore, on QoL. Although the authors conducted a case-control study, they administered the PDQ-8 by telephone only to the PD patients' group during the last days of the first Italian lockdown. They observed a low impact on QoL, suggesting a minimal impact on the perception of the QoL. It should be noted that results should be interpreted with caution due to the small sample.

In another study, Subramanian et al. (2020) developed a specific survey to examine the general perception of QoL on 1,451 PD patients, including the items from the Patient-Reported Outcomes Measurement Information System. Findings suggested that social isolation was associated in a statistical significance range with PD severity, measured by the PRO-PD, and worse QoL (*p* < 0.01). Moreover, the latter correlated with loneliness and worse social health: patients reported a worsening in their social health and in social satisfaction and performance, while symptoms severity increased. These results confirmed that worse QoL is associated with both progression of disease symptoms and loneliness.

In another observational study based on PDQ-8, Fabbri et al. (2021) recorded a negative impact of lockdown on QoL in about half of the cohort. Based on the patients' perception assessed by a PGI-I scale, a correlation was found between worse QoL and severe worsening clinical symptoms (i.e., at least 5 reported aggravated domains). QoL worsened as the severity of motor and NMS increased, which is unsurprisingly and probably related to the first COVID-19 self-isolation, the imposed social distancing, and the abrupt onset of the lockdown but also of the pandemic itself. The concomitant aggravation of motor symptoms, such as tremor and rigidity, which are particularly sensitive to stress, may be related to increased anxiety/depression as well as to the diffuse interruption of physiotherapy and any outdoor physical activities, all factors that are widely recognized as having a higher negative impact on QoL.

Similarly, in a study by Saluja et al. (2021) conducted between June and September 2020 on Indian patients, an association between NMS severity and QoL was found. All participants were interviewed on the telephone according to the PDQ-8 and the NMSS. Over the course of the lockdown period, an increase in NMS (mood changes, insomnia, and pain, which were observed, respectively, in 42.3, 34.6, and 34.6% of patients) was registered, as well as of the total NMSS score (in the last 4 weeks), as reported by patients. In line with the previous studies, worsening of NMS during the lockdown became an independent predictor of poor QoL among patients with PD. Moreover, a selective isolation from other family members was proven to have deleterious impact on their QoL.

In addition to the objective data measured with standardized and validated scales, a useful insight comes from studies that investigated patients' subjective perception of lockdown-related change in QoL through *ad hoc* questionnaires (Subramanian et al., 2020; Kumar et al., 2021; Silva-Batista et al., 2021; Templeton et al., 2021). However, these studies as well-reported discrepant results.

In Kumar et al. (2021), more than a half of the PD patients (*n* = 451, 54.2%) who replied to a single-question on an *ad hoc* questionnaire reported that they were unsatisfied with their QoL during home confinement, especially due to worsening of both motor and NMS (*n* = 320, 38.5%), and fear of contracting COVID-19 (*n* = 162, 19.5%). Among patients who reported dissatisfaction with their QoL, almost half of them were patients who had reported new onset or worsening of sleep disturbances related to isolation. In line with these data, a statistically significant association between poorer QoL and the new onset or worsening of sleep disturbances was found (*p* < 0.001). Thus, sleep may have had an impact on the QoL of these patients.

The results of another study (Silva-Batista et al., 2021), conducted by administering to Brazilian PD patients a questionnaire by telephone about the perception of negative changes in QoL during social distancing, showed that half of the participants reported low PDQ-8 scores (49.7%), indicating a good QoL, and almost one third (30.3%) reported no variation. Further analysis showed that the self-reported decline in the QoL was a common predictor of self-reported clinical worsening, i.e., MDS-UPDRS part IB, MDS-UPDRS part II, and emotional and mental health scores. Therefore, the subjects' perception of a worsened QoL had also negatively affected the perception of the motor and non-motor aspects of daily life and of their own emotional and mental health status.

In Templeton et al. (2021), PD patients were asked to reply to a survey about their QoL to assess when they felt their best and how various factors influenced that feeling, such as the time of the day and typical daily events or settings. In terms of hours, results showed that participants felt their best in the morning, then the sensation progressively faded and at evenings they felt their worst. In terms of typical events or settings, this feeling of wellbeing grew after engaging in physical activity (*n* = 19, 67.86%) or in comfortable environments (*n* = 17, 60.71%). Authors also found that per-day minutes of activity and the number of total activities decreased significantly (*p* = 0.022) due to COVID-19 restrictions, and that 82.14% (*n* = 23) of subjects self-reported that at least one symptom had worsened from moderate to severe. The authors argued that not being able to participate in structured in-person programs because of lockdown had a direct negative effect on patients' overall activity levels and disease progression, which in turn affected their QoL: such programs provide an opportunity to exercise and find a supportive environment that promotes social relationships, both relevant factors for wellbeing.

In a study conducted in the Netherlands by Dommershuijsen et al. (2021), the authors investigated in a cohort of PD patients the impact of COVID-19 stressors on mental health using a questionnaire, and also assessed their QoL with PDQ-39. An association was observed between stressors sum score (95% CI: 0.02–0.04), social stressors (beta: 0.06, 95% CI: 0.04–0.08), care stressors (beta: 0.05, 95% CI: 0.02–0.08), and the PDQ-39 score. Furthermore, patients who perceived COVID-19 stressors as “troublesome” also had a worse QoL.

Lastly, Krzysztoń et al. (2022) studied a sample of 47 PD patients through an online survey regarding differences in QoL during the pandemic, to which a considerable amount of respondents reported a decrease of 1 (*n* = 22, 47%), 2 (*n* = 9, 19%), or 3 (*n* = 2, 4%) points (on a scale from 1, signifying a very poor QoL, to 5, indicating a very good QoL, evaluating changes between pre-pandemic times and the survey completion). Patients suffering from PD for over 5 years experienced a significantly greater decrease in QoL compared to patients with a shorter history of the disease. Furthermore, a statistically significant association was found between changes in QoL and feelings of isolation (*p* = 0.009) and reduced social contact (*p* = 0.022).

In conclusion, the studies presented in this section highlight a direct and indirect impact of lockdown for COVID-19 on the QoL of PD patients.

## Discussion

The purpose of this paper was to perform a critical review of the literature that investigated the psychological impact of COVID-19 and restrictive measures on patients with PD. We therefore focused in particular on psychological aspects such as depression and anxiety, as well as on indirect measures such as the impact on sleep and QoL.

The results on depression showed that, when comparing data collected during the pandemic and before, there was not a statistically significant impact on patients (El Otmani et al., 2021; Falla et al., 2021; HØrmann Thomsen et al., 2021). However, the pre-pandemic evaluations often date back to up to 2 years, therefore it is difficult to exclude that other variables may have intervened (HØrmann Thomsen et al., 2021). Data are more heterogeneous when patients are compared with a control group. In fact, while the comparison with healthy subjects reveals statistically significant differences in depressive symptoms (Xia et al., 2020). A greater deviation in mood in PD patients than in the general population is in line with the literature (Reijnders et al., 2008) and could be partially explained by the pathophysiology of PD, which naturally increases the risk of mood deviation as reduced dopamine levels compromise coping mechanisms for depression (Marsh, 2013; Helmich and Bloem, 2020). Conversely, when the participants' family members or caregivers belong to the control group, the differences become minimal (Oppo et al., 2020; Suzuki et al., 2021). Only one study fails to show a statistically significant difference when comparing patients with healthy subjects (Balci et al., 2021). These results highlight how the patient's emotional load has important psychological repercussions also on family members and caregivers.

The literature shows that PD patients reported a growing presence of anxiety symptoms during isolation both over the course of time (compared with pre-lockdown data) (Falla et al., 2021; HØrmann Thomsen et al., 2021) and against other control groups (caregivers and healthy subjects). Overall, from a qualitative point of view, this overview indicated that PD patients show higher anxiety levels than control groups, which can either include caregivers or healthy controls. However, only two studies were able to find a statistically significant difference (Salari et al., 2020; Shalash et al., 2020), whereas the remaining studies reported no differences between PD and other control groups (Oppo et al., 2020; Xia et al., 2020; Balci et al., 2021; Kitani-Morii et al., 2021; Suzuki et al., 2021). Therefore, although the reduction of physical and social activity as well as the dopamine-related failure to adapt may be a vulnerability factor in coping with stress during the COVID-19 pandemic (HØrmann Thomsen et al., 2021; Kumar et al., 2021), in most of the studies reviewed the results of the assessments anxiety levels were not statistically significant. These findings must be interpreted carefully in the light of the studies' limitations, both in methodology and design. From a longitudinal perspective, there are several ways in which time can be factored into the design of studies: the aforementioned authors have variously compared anxiety levels registered before or at the beginning of the restrictions and during or after the 2020 lockdown period. The absence of pre-lockdown anxiety levels measures impedes the interpretation of the effect direction. In fact, reverse causation could importantly influence the explanation of results, as patients with pre-existing anxiety symptoms might have reported a greater influence of COVID-19 stressors on their mental health.

Findings from studies on sleep disturbances are also heterogeneous. However, as for other of the examined topics, PD patients showed a greater probability of suffering from sleep disorders than healthy subjects, while no statistically significant difference emerged from the comparison with family members (Kitani-Morii et al., 2021), thus confirming the heavy psychological burden experienced by caregivers and already detected by previous studies. Few articles indicate a subjective worsening of sleep quality or the onset of sleep disturbances during isolation (Guo et al., 2020; Janiri et al., 2020; Song et al., 2020; de Rus Jacquet et al., 2021; Fabbri et al., 2021; Kumar et al., 2021; Saluja et al., 2021; Suzuki et al., 2021; Templeton et al., 2021). Overall, across the studies, only a minority of PD patients reported changes in sleep quality. The moderate worsening of sleep quality, with no pre-lockdown available data, could indicate that restrictions did not have a drastic effect on sleep.

Finally, various published studies focused on the impact of COVID-19 restrictive measures on the QoL in PD patients. Results are mixed and heterogeneous, probably due to the complex effects of the pandemic on the motor and NMS in PD patients. In fact, several studies showed a post-lockdown worsening in PD patients (Guo et al., 2020; Song et al., 2020), while another group of studies showed an unexpected improvement (HØrmann Thomsen et al., 2021) or no variation in the QoL of PD patients compared to pre-lockdown (Falla et al., 2021; Luis-Martínez et al., 2021). A possible explanation is that researchers assessed QoL in distinct time points, resorting to different measures that have been used in clinical settings or research, or as an indirect measure from other studies. Despite some variations in results, compared with control groups composed by healthy subjects, caregivers, and patients with other neurological conditions, PD patients showed a statistically significant deterioration of QoL (Shalash et al., 2020; Sahin et al., 2021; Suzuki et al., 2021). Considering the highly disabling nature of PD, it is logically arguable that restrictions might have had a greater impact on PD patients due to the inability to move, exercise, maintain social relationships, participate in support groups, and preserve daily routines.

### Limitation

The selected papers for this work have limitations that are worth consideration. First of all, the data presented by the studies are only preliminary findings, as most of them relate to the first wave of COVID-19. Secondly, we have selected the articles from a single database and only in English, so we cannot exclude that other studies have shown different results. Moreover, an important limitation is that most studies did not consider the potential effect of disease duration and symptom severity on changes in NMS. In regard to experimental designs, several studies based on groups and/or on a single assessment during or after the lockdown are marked by a lack of pre-pandemic data; vice-versa, in longitudinal studies a control group is often missing. As a result, it is difficult to determine whether the detected changes are due to restrictions or to disease progression. In addition, some studies have small normative samples and, therefore, are not representative. The heterogeneity of samples across studies does not allow for accurate comparison of results, which is also made difficult by the different restrictions in place in different countries.

## Conclusion

In conclusion, the data summarized in this review suggest that the restrictions adopted by different countries to contrast COVID-19 have led to a negative psychological impact on PD patients. The pandemic, which is currently still ongoing, may have also caused additional changes on NMS of PD. Thus, in the future, it would be interesting to conduct further longitudinal studies, to follow the long-term impact of restrictions implemented in subsequent COVID-19 waves on PD patients.

This review emphasizes the importance of providing ongoing healthcare to monitor and support the mental health of PD patients during a public health emergency or in any situation of forced social isolation. This can be accomplished by implementing the use of telehealth and complementary resource such as peer support that can improved relationships with providers, care engagement, and various recovery-related outcomes in people with chronic illness (Suresh et al., 2021).

## Data availability statement

The original contributions presented in the study are included in the article/Supplementary Materials, further inquiries can be directed to the corresponding author.

## Author contributions

FM and FR conceptualized the study. EZ, BC, and DM edited the data. FR, EZ, RF, and FM contributed to methodology. SB supervised the drafting of the manuscript and acquired the funds. FR, FM, EZ, BC, and DM wrote the first draft of the manuscript. FR, FM, EZ, GF, and AD revised and edited the manuscript. All authors contributed to the article and approved the submitted version.

## Funding

Open Access article charges are covered by Ricerca Corrente (IRCCS RC-2022 Grant no. 01) from the Italian Ministry of Health.

## Conflict of interest

The authors declare that the research was conducted in the absence of any commercial or financial relationships that could be construed as a potential conflict of interest.

## Publisher's note

All claims expressed in this article are solely those of the authors and do not necessarily represent those of their affiliated organizations, or those of the publisher, the editors and the reviewers. Any product that may be evaluated in this article, or claim that may be made by its manufacturer, is not guaranteed or endorsed by the publisher.

## References

[B1] Balci B. Aktar B. Buran S. Tas M. Donmez Colakoglu B. (2021). Impact of the COVID-19 pandemic on physical activity, anxiety, and depression in patients with Parkinson's disease. Int. J. Rehabil. Res. 44, 173–176. 10.1097/mrr.0000000000000460 33653991 PMC8103842

[B2] Bloem B.R. Dorsey E.R. Okun M.S. (2020). The coronavirus disease 2019 crisis as catalyst for telemedicine for chronic neurological disorders. JAMA Neurol. 77, 927–928. 10.1001/jamaneurol.2020.1452 32329796

[B3] Boman K. (1971). Effect of emotional stress on spasticity and rigidity. J. Psychosom. Res. 15, 107–112. 10.1016/0022-3999(71)90079-1 5576337

[B4] Brown E.G. Chahine L.M. Goldman S.M. Korell M. Mann E. Kinel D.R. . (2020). The effect of the COVID-19 pandemic on people with Parkinson's disease. J. Parkinsons. Dis. 10, 1365–1377. 10.3233/JPD-202249 32925107 PMC7683050

[B5] Corti E.J. Johnson A.R. Gasson N. Bucks R.S. Thomas M.G. Loftus A.M. (2018). Factor structure of the ways of coping questionnaire in Parkinson's disease. Parkinsons. Dis. 2018, 7128069. 10.1155/2018/7128069 30631419 PMC6304892

[B6] de Rus Jacquet A. Bogard S. Normandeau C.P. Degroot C. Postuma R.B. Dupré N. . (2021). Clinical perception and management of Parkinson's disease during the COVID-19 pandemic: a Canadian experience. Parkinsonism Relat. Disord. 91, 66–76. 10.1016/j.parkreldis.2021.08.018 34536727 PMC8407944

[B7] Del Prete E. Francesconi A. Palermo G. Mazzucchi S. Frosini D. Morganti R. . (2021). Prevalence and impact of COVID-19 in Parkinson's disease: evidence from a multi-center survey in Tuscany region. J. Neurol. 268, 1179–1187. 10.1007/s00415-020-10002-6 32880722 PMC7471534

[B8] Dommershuijsen L.J. Van der Heide A. Van den Berg E.M. Labrecque J.A. Ikram M.K. Ikram M.A. . (2021). Mental health in people with Parkinson's disease during the COVID-19 pandemic: potential for targeted interventions? NPJ Parkinsons Dis. 7, 95. 10.1038/s41531-021-00238-y 34711842 PMC8553848

[B9] El Otmani H. El Bidaoui Z. Amzil R. Bellakhdar S. El Moutawakil B. Abdoh Rafai M. (2021). No impact of confinement during COVID-19 pandemic on anxiety and depression in Parkinsonian patients. Rev. Neurol. 177, 272–274. 10.1016/j.neurol.2021.01.005 33610345 PMC7877213

[B10] Fabbri M. Leung C. Baille G. Béreau M. Brefel Courbon C. Castelnovo G. . (2021). A French survey on the lockdown consequences of COVID-19 pandemic in Parkinson's disease. The ERCOPARK study. Parkinsonism Relat. Disord. 89, 128–133. 10.1016/j.parkreldis.2021.07.013 34293534 PMC9272278

[B11] Falla M. Dodich A. Papagno C. Gober A. Narduzzi P. Pierotti E. . (2021). Lockdown effects on Parkinson's disease during COVID-19 pandemic: a pilot study. Acta Neurol. Belg. 121, 1191–1198. 10.1007/s13760-021-01732-z 34212285 PMC8248756

[B12] Fasano A. Antonini A. Katzenschlager R. Krack P. Odin P. Evans A.H. . (2020). Management of advanced therapies in Parkinson's disease patients in times of humanitarian crisis: the COVID-19 experience. Mov. Disord. Clin. Pract. 7, 361–372. 10.1002/mdc3.12965 32373652 PMC7197306

[B13] Frazier L.D. (2000). Coping with disease-related stressors in Parkinson's disease. Gerontologist 40, 53–63. 10.1093/geront/40.1.53 10750313

[B14] Garg D. Dhamija R.K. (2020). The challenge of managing Parkinson's disease patients during the COVID-19 pandemic. Ann. Indian Acad. Neurol. 23(Suppl. 1), S24–S27. 10.4103/aian.AIAN_295_20 32419750 PMC7213030

[B15] Guo D. Han B. Lu Y. Lv C. Fang X. Zhang Z. . (2020). Influence of the COVID-19 pandemic on quality of life of patients with Parkinson's disease. Parkinsons. Dis. 2020, 1216568. 10.1155/2020/1216568 33062247 PMC7537675

[B16] Haahr A. Kirkevold M. Hall E.O. Ostergaard K. (2011). Living with advanced Parkinson's disease: a constant struggle with unpredictability. J. Adv. Nurs. 67, 408–417. 10.1111/j.1365-2648.2010.05459.x 20946567

[B17] Hall M.E. Church F.C. (2020). Exercise for older adults improves the quality of life in Parkinson's disease and potentially enhances the immune response to COVID-19. Brain Sci. 10, 612. 10.3390/brainsci10090612 32899958 PMC7563553

[B18] Helmich R.C. Bloem B.R. (2020). The impact of the COVID-19 pandemic on Parkinson's disease: hidden sorrows and emerging opportunities. J. Parkinsons. Dis. 10, 351–354. 10.3233/JPD-202038 32250324 PMC7242824

[B19] HØrmann Thomsen T. Wallerstedt S.M. Winge K. Bergquist F. (2021). Life with Parkinson's disease during the COVID-19 pandemic: the pressure is “OFF”. J. Parkinsons. Dis. 11, 491–495. 10.3233/jpd-202342 33459663

[B20] Janiri D. Petracca M. Moccia L. Tricoli L. Piano C. Bove F. . (2020). COVID-19 pandemic and psychiatric symptoms: the impact on Parkinson's disease in the elderly. Front. Psychiatry 11, 581144. 10.3389/fpsyt.2020.581144 33329124 PMC7728715

[B21] Kitani-Morii F. Kasai T. Horiguchi G. Teramukai S. Ohmichi T. Shinomoto M. . (2021). Risk factors for neuropsychiatric symptoms in patients with Parkinson's disease during COVID-19 pandemic in Japan. PLoS ONE 16, e0245864. 10.1371/journal.pone.0245864 33481879 PMC7822544

[B22] Knapik A. Szefler-Derela J. Wasiuk-Zowada D. Siuda J. Krzystanek E. Brzek A. (2021). Isolation related to the COVID-19 pandemic in people suffering from Parkinson's disease and activity, self-assessment of physical fitness and the level of affective disorders. Healthcare 9, 1562. 10.3390/healthcare9111562 34828608 PMC8624023

[B23] Krzysztoń K. Mielańczuk-Lubecka B. Stolarski J. Poznańska A. Kepczyńska K. Zdrowowicz A. . (2022). Secondary impact of COVID-19 pandemic on people with Parkinson's disease-results of a polish online survey. Brain Sci. 12, 26. 10.3390/brainsci12010026 35053770 PMC8774235

[B24] Kumar N. Gupta R. Kumar H. Mehta S. Rajan R. Kumar D. . (2021). Impact of home confinement during COVID-19 pandemic on sleep parameters in Parkinson's disease. Sleep Med. 77, 15–22. 10.1016/j.sleep.2020.11.021 33302094 PMC7682933

[B25] Luis-Martínez R. Di Marco R. Weis L. Cianci V. Pistonesi F. Baba A. . (2021). Impact of social and mobility restrictions in Parkinson's disease during COVID-19 lockdown. BMC Neurol. 21, 332. 10.1186/s12883-021-02364-9 34461838 PMC8404403

[B26] Macht M. Kaussner Y. Möller J.C. Stiasny-Kolster K. Eggert K.M. Krüger H.P. . (2007). Predictors of freezing in Parkinson's disease: a survey of 6,620 patients. Mov. Disord. 22, 953–956. 10.1002/mds.21458 17377927

[B27] Marsh L. (2013). Depression and Parkinson's disease: current knowledge. Curr. Neurol. Neurosci. Rep. 13, 409. 10.1007/s11910-013-0409-5 24190780 PMC4878671

[B28] Miocinovic S. Ostrem J.L. Okun M.S. Bullinger K.L. Riva-Posse P. Gross R.E. . (2020). Recommendations for deep brain stimulation device management during a pandemic. J. Parkinsons. Dis. 10, 903–910. 10.3233/JPD-202072 32333552 PMC7458514

[B29] Montanaro E. Artusi C.A. Rosano C. Boschetto C. Imbalzano G. Romagnolo A. . (2022). Anxiety, depression, and worries in advanced Parkinson disease during COVID-19 pandemic. Neurol. Sci. 43, 341–348. 10.1007/s10072-021-05286-z 33948763 PMC8096160

[B30] Oppo V. Serra G. Fenu G. Murgia D. Ricciardi L. Melis M. . (2020). Parkinson's disease symptoms have a distinct impact on caregivers' and patients' stress: a study assessing the consequences of the COVID-19 lockdown. Mov. Disord. Clin. Pract. 7, 865–867. 10.1002/mdc3.13030 33043088 PMC7533970

[B31] Papa S.M. Brundin P. Fung V.S.C. Kang U.J. Burn D.J. Colosimo C. . (2020). Impact of the COVID-19 pandemic on Parkinson's disease and movement disorders. Mov. Disord. 35, 711–715. 10.1002/mds.28067 32250460 PMC7996401

[B32] Reijnders J.S. Ehrt U. Weber W.E. Aarsland D. Leentjens A.F. (2008). A systematic review of prevalence studies of depression in Parkinson's disease. Mov. Disord. 23, 183–189; quiz 313. 10.1002/mds.21803 17987654

[B33] Robbins T.W. Cools R. (2014). Cognitive deficits in Parkinson's disease: a cognitive neuroscience perspective. Mov. Disord. 29, 597–607. 10.1002/mds.25853 24757109

[B34] Sahin S. Karsidag S. Cinar N. Ates M.F. Demir S. Eren F. . (2021). The impact of the COVID-19 lockdown on the quality of life in chronic neurological diseases: the results of a COVQoL-CND study. Eur. Neurol. 84, 450–459. 10.1159/000517380 34344010 PMC8450832

[B35] Salari M. Zali A. Ashrafi F. Etemadifar M. Sharma S. Hajizadeh N. . (2020). Incidence of anxiety in Parkinson's disease during the coronavirus disease (COVID-19) pandemic. Mov. Disord. 35, 1095–1096. 10.1002/mds.28116 32395849 PMC7273007

[B36] Saluja A. Parihar J. Garg D. Dhamija R.K. (2021). The impact of COVID-19 pandemic on disease severity and quality of life in Parkinson's disease. Ann. Indian Acad. Neurol. 24, 217–226. 10.4103/aian.AIAN_1240_20 34220066 PMC8232490

[B37] Shalash A. Roushdy T. Essam M. Fathy M. Dawood N.L. Abushady E.M. . (2020). Mental health, physical activity, and quality of life in Parkinson's disease during COVID-19 pandemic. Mov. Disord. 35, 1097–1099. 10.1002/mds.28134 32428342 PMC7276909

[B38] Silva-Batista C. Coelho D.B. Júnior R.C.F. Almeida L.R. Guimarães A. Nóbrega K.C.C. . (2021). Multidimensional factors can explain the clinical worsening in people with Parkinson's disease during the COVID-19 pandemic: a multicenter cross-sectional trial. Front. Neurol. 12, 708433. 10.3389/fneur.2021.708433 34393984 PMC8362931

[B39] Song J. Ahn J.H. Choi I. Mun J.K. Cho J.W. Youn J. (2020). The changes of exercise pattern and clinical symptoms in patients with Parkinson's disease in the era of COVID-19 pandemic. Parkinsonism Relat. Disord. 80, 148–151. 10.1016/j.parkreldis.2020.09.034 33002722 PMC7510770

[B40] Subramanian I. Farahnik J. Mischley L.K. (2020). Synergy of pandemics-social isolation is associated with worsened Parkinson severity and quality of life. NPJ Parkinsons Dis. 6, 28. 10.1038/s41531-020-00128-9 33083522 PMC7545190

[B41] Suresh R. Alam A. Karkossa Z. (2021). Using peer support to strengthen mental health during the COVID-19 pandemic: a review. Front. Psychiatry 12, 714181. 10.3389/fpsyt.2021.714181 34322045 PMC8310946

[B42] Suzuki K. Numao A. Komagamine T. Haruyama Y. Kawasaki A. Funakoshi K. . (2021). Impact of the COVID-19 pandemic on the quality of life of patients with Parkinson's disease and their caregivers: a single-center survey in Tochigi prefecture. J. Parkinsons. Dis. 11, 1047–1056. 10.3233/jpd-212560 33780375

[B43] Templeton J.M. Poellabauer C. Schneider S. (2021). Negative effects of COVID-19 stay-at-home mandates on physical intervention outcomes: a preliminary study. J. Parkinsons. Dis. 11, 1067–1077. 10.3233/jpd-212553 33867363

[B44] van der Heide A. Meinders M.J. Bloem B.R. Helmich R.C. (2020). The impact of the COVID-19 pandemic on psychological distress, physical activity, and symptom severity in Parkinson's disease. J. Parkinsons. Dis. 10, 1355–1364. 10.3233/JPD-202251 32925108 PMC7683090

[B45] Weintraub D. Burn D.J. (2011). Parkinson's disease: the quintessential neuropsychiatric disorder. Mov. Disord. 26, 1022–1031. 10.1002/mds.23664 21626547 PMC3513835

[B46] Xia Y. Kou L. Zhang G. Han C. Hu J. Wan F. . (2020). Investigation on sleep and mental health of patients with Parkinson's disease during the Coronavirus disease 2019 pandemic. Sleep Med. 75, 428–433. 10.1016/j.sleep.2020.09.011 32980664 PMC7481072

[B47] Zach H. Dirkx M. Bloem B.R. Helmich R.C. (2015). The clinical evaluation of Parkinson's tremor. J. Parkinsons. Dis. 5, 471–474. 10.3233/JPD-150650 26406126 PMC4923747

