# Supplementary material for: Changes in non-motor symptoms in patients with Parkinson's disease following COVID-19 pandemic restrictions: A systematic review
Source: Front Psychol. 2022 Jul 22;13:939520. doi: 10.3389/fpsyg.2022.939520 (PMC9355666; doi:10.3389/fpsyg.2022.939520)
Supplement: Supplementary file 1 [file Table_1.docx]

***Supplementary Material***

**Table 1.** Overview of selected studies.

| **Author and year** | **Country** | **Subjects** | **Age (years)** | **PD duration (years)** | **Study design** | **Data collection time point(s)** | **Assessment tool** | **Outcome measure** | **Main results** |
| --- | --- | --- | --- | --- | --- | --- | --- | --- | --- |
| Guo et al. (2020) | China | 113 PD (66 M, 47 F) | 69.5±7.8^a^ | 6.05±0.8^a^ | Longitu  dinal study | T0: February 1-March 31, 2020 (during restrictions)  T1: April 1-30, 2020 (after restrictions) | Survey (T0)  PDQ-39 (T0, T1) | Depression  Sleep  QoL | Data for 108 PD were collected.  79.6% self- reported new/worsening symptoms:  depression 50%, sleep disorders 39.8%.  Worse QoL T0 vs T1 (p<0.001)  mainly in PD who had difficulties in getting doctor’s advice and PD who changed routine medications. |
| Janiri et al. (2020) | Italy | 134 PD  (NR) | NR | NR | Cross-sectional study | April 1-15, 2020 (during restrictions) | Survey | Depression  Sleep | 75.4% reported lifetime psychiatric symptoms.  22.8% self-reported worsening of psychiatric symptoms:  depression 82.6%, insomnia 52.2%, RBD 21.7%. |
| Oppo et al. (2020) | Italy | 32 PD  (24 M, 8 F)  32 Caregivers (6 M, 26 F) | PD: 72.5±8.7^a^  Caregivers: 64.6±10.3^a^ | 11.6±4.5^a^ | Cross-sectional study | Last 10 days of first 2020 lockdown (may 2020) | HADS  NMSS (PD)  PDQ-8 (PD) | Depression  Anxiety  NMS  QoL | No differences in depression (p=0.126) and anxiety scores (p=0.808) in PD vs Caregivers.  Worse anxiety score in PD not engaged in home physical activity (p=0.044) and PD with higher stress levels (p=0.006).  Worse mood/cognition subscale score in PD with higher stress levels (p<0.05).  Low impact of restrictions on QoL. |
| Salari et al. (2020) | Iran | 137 PD (47 M, 90 F)  95 Caregivers (24 M, 71 F)  442 HC  (NR) | PD: 55±10.7^a^  Caregivers: 43±9.3^a^  HC: NR | NR | Case-control study | NR | BAI-II-Persian version | Anxiety | Worse anxiety score in PD vs Caregivers and vs HC (p<0.001). |
| Shalash et al. (2020) | Egypt | 38 PD (29 M, 9 F)  20 CG (14 M, 6 F) | PD: 55.6±9.9^a^  CG: 55.5±5.7^a^ | 4.7±3.2^a^ | Case-control study | NR | DASS-21  Ad hoc questionnaire  PDQ-39 | Depression  Anxiety  Anxiety  QoL | Higher prevalence and worse score for depression (p=0.015) and anxiety (p=0.001) in PD vs CG.    52.6% PD self-reported anxiety/stress due to COVID-19.  Worse QoL in PD vs CG (p<0.001). |
| Song et al. (2020) | Republic of Korea | 100 PD (54 M, 46 F) | 70 (62.3-76)^b^ | 6 (4-10)^b^ | Longitu  dinal study | T0: December 2019-January 20, 2020 (before COVID-19 pandemic)  T1: May 1-20, 2020 (during restrictions) | Ad hoc questionnaire (T1)  SE-ADL (T0; T1) | Depression  Sleep  QoL | Perception of worsened NMS in 27 PD:  depression 5%, sleep reduction 5%.  Worse SE-ADL score T0 vs T1 (p=0.031) in PD. |
| Subramanian et al. (2020) | USA | 1527 PD (677 M, 839 F and 11 NR) | 62.9±9.2^a^ | 4.8±4.9^a^ | Cross-sectional study | NR | PROMIS Global of QoL | QoL | Data for QoL of 1451 PD (95%) were collected.  Lonely PD showed greater symptom severity than not lonely PD (p<0.01) and rated lower QoL.  PD who documented having a lot of friends had 21% fewer symptoms than those with few or no friends (p<0.01). |
| van der Heide et al. (2020) | the Netherlands | 358 PD (220 M, 138 F) | 62.8±9^a^ | 3.9±1.8^a^ | Longitudinal study | T0: visit 7.5 months before T1 (before COVID-19)  T1: April 21-May 25, 2020 (during restrictions) | STAI (T0)  PAS Part B (T1) | Anxiety  Episodic anxiety | High anxiety score at T0.  High episodic anxiety score at T1.  Higher episodic anxiety score at T1 and higher anxiety at T0 predicted increased psychological distress during the pandemic.  COVID-19 pandemic leads to a worsening of PD symptoms by evoking psychological distress as well as reduced physical activity. |
| Xia et al.  (2020) | China | 119 PD (61 M, 58 F)  169 HC (76 M, 93 F) | PD: 61.2±8.8^a^  HC: 59.8±8.1^a^ | 6.84±4.6^a^ | Case-control study | April 20-30, 2020 with reference to February-April 2020 (during restrictions) | PSQI  HADS | Sleep  Depression  Anxiety | PD had significantly higher PSQI score and higher prevalence of sleep disturbance vs HC (p<0.001).  Worse depression score in PD vs HC (p=0.022), mainly in PD with poor sleep (p=0.002).  No differences in prevalence of anxiety (p=0.410) and anxiety score in PD vs HC (p=0.579).  Higher prevalence (p=0.038) and worse score (p<0.001) of anxiety in PD with poorer sleep. |
| Balci et al. (2021) | Turkey | 45 PD (30 M, 15 F)  43 HC (24 M, 19 F) | PD: 67 (60-73.5)^b^  HC: 66 (58-71)^b^ | 8 (5-10)^b^ | Case control study | June 15-20, 2020 with reference to March 11-June 1, 2020 (during restrictions) | HADS  Ad hoc questionnaire | Depression  Anxiety  Sleep | No PD and HC had depression (p=0.524) or anxiety (p=0.977).  Worsened sleep in 7 PD≥65 yr and 2 PD<65 yr.  Worsened daytime sleepiness in 6 PD≥65 yr and 1 PD<65 yr. |
| de Rus Jacquet et al. (2021) | Canada | 417 PD (256 M, 161 F) | 68.2±9.7^a^ | 8±5.4^a^ | Cross-sectional study | May 20-September 16, 2020 with reference to mid March, 2020 (start of restrictions) | Survey | Depression  Anxiety  Sleep | Depression  56 PD reported changes: 67.86% PD worsened, 32.14% had day to day change, none improved.  Anxiety  125 PD reported changes: 75.2% worsened, 1.6% improved, 23.2% had day to day change.  Sleep  63% PD no perceived changes in sleep, 8.39% improved, 28% worsened. |
| Del Prete et al. (2021) | Italy | 740 PD  (NR) | NR | NR | Cross-sectional study | April 10-May 4, 2020 (during restrictions) | Survey    DASS-21 | Depression  Anxiety  Sleep  Depression  Anxiety | Data for 733 PD were collected.  No worsening in mood score 74.9%.  No worsening in anxiety score 74.6%.  No new/worsening in insomnia score 77.4%.  Data for 120 PD were collected.  Total DASS-21 score (z score=0.57). |
| Dommershuijsen et al. (2021) | the Netherlands | 844 PD (523 M, 321 F) | 70.3±7.8^a^ | 6.4±5.4^a^ | Cross-sectional study | April 14, 2020- February 25, 2021 (during COVID-19 pandemic) | BDI  STAI  PDQ-39 | Depression  Anxiety  QoL | Depression score increased as perceived  COVID-19 stressors increased, mainly in women and PD with more severe symptoms.  Anxiety score increased as perceived COVID-19 stressors increased, mainly in women, PD with more severe symptoms, disease duration >5 yr. and highly educated.  QoL score increased as perceived COVID-19 stressors increased. |
| El Otmani et al. (2021) | Morocco | 50 PD  (24 M, 26 F) | 60.4±10.4^a^ | NR | Longitudinal study | T0: March 16, 2020 (start of restrictions)  T1: after 6 weeks of restrictions | HADS (T0, T1)  Interview (T1) | Depression  Anxiety  Depression  Anxiety | No differences in depression (p=0.14) and anxiety (p=0.44) scores T0 vs T1.  Supporting factors:  40% the presence of relatives.  Unsupportive factors:  33,3% the reduced physical activity;  14% lack of family support. |
| Fabbri et al. (2021) | France | 2653 PD (1459 M, 1194 F) | NR | NR | Cross-sectional study | March 16-May 16, 2020 with reference to mid March-mid April, 2020 (during restrictions) | Survey  PGI-I  PDQ-8 | Depression  Anxiety  Sleep  Depression  Anxiety  QoL | 40.9% of PD reported a worsening of symptoms.  Perception of new/worsened symptoms: depression 3.4%, anxiety 6.1%, sleep disorders 4.1%.  Perception of worsened psychic state domain (anxiety and depression) 46.3 %.  Moderate impact of restrictions on QoL. Worse QoL in PD with more aggravated disease (p<0.0001). |
| Falla et al. (2021) | Italy | 14 PD  (7 M, 7 F) | 64.9±8.5^a^ | 5.7±4.1^a^ | Longitudinal study | T0: February 2020 (before COVID-19 pandemic)  T1: April 24-May 1, 2020 (during restrictions) | GDS  OR-PAS  MDS-UPDRS-Part I  PDQ-39 | Depression  Anxiety  Sleep  QoL | 35.7% of PD presented depression at T0 and 28.6% at T1.  No significant differences in depression score T0 vs T1 (p=1.000).  Higher prevalence (78.6%) and worse total score of anxiety T1 vs T0 (p=0.007), also due to avoidance behaviour subscale worsening (p<0.001).  Subjective worsening of sleep problems in 5 PD and daytime sleepiness in 8 PD at T1.  No differences in QoL T0 vs T1 (p=0.221). |
| HØrmann Thomsen et al. (2021) | Denmark and Sweden | 67 PD  (35 M, 32 F) | 70 (IQR NR)^b^ | 6 (IQR NR)^b^ | Longitudinal study | T0: August-November, 2018 (before COVID-19 pandemic)  T1: April-June, 2020 (during restrictions) | PRO-PD  PDQ-8  BDI-II  PDQ-39 | Depression  Anxiety  Sleep  QoL  Depression  Anxiety (1 item)  QoL  Sleep (1 item) | Data for 33 Swedish PD were collected.  No differences in depression score T0 vs T1 (p=0.129).  Worse anxiety score T1 vs T0 (p=0.035).  Improved sleep score T1 vs T0 (p=0.039).  Improved QoL T1 vs T0 (p=0.027).  Data for 34 Danish PD were collected.  No difference in depression score T0 vs T1 (p=0.981).  Worse anxiety score T1 vs T0 (p=0.001).  Improved QoL score T1 vs T0 (p=0.018).  No differences in sleep problems score T0 vs T1 (p=0.253). |
| Kitani-Morii et al. (2021) | Japan | 39 PD (25 M, 14 F)  32 CG (5 M, 27 F) | PD: 72.3±10.9^a^  CG: 66.4±13.8^a^ | PD <5: 22 ±56.4^a^  PD ≥5: 17 ± 43.5^a^ | Cross-sectional study | April 22-May 15, 2020 (during restrictions) | PHQ-9  GAD-7  ISI  Interview | Depression  Anxiety  Sleep  Anxiety  Sleep | Data on depression of 38 PD and 31 CG were collected.  Higher score (p=0.01) and prevalence (p=0.002) of clinical depression in PD using PHQ-9 (39%) vs CG (6%).  No differences in prevalence and score in clinical anxiety in PD (48%) vs CG (34%) (p=0.223).  No differences in prevalence and score in clinical insomnia in PD (33%) vs CG (21%) (p=0.286).  No difference in subjective worsening of anxiety (p=0.25) and sleep (p=0.64) PD vs CG. |
| Knapik et al. (2021) | Poland | 30 PD (18 M, 12 F) | 69.7±7.9^a^ | 7.68± 5^a^ | Longitudinal study | T0: few months before COVID-19 pandemic  T1: 90 days after restrictions | Ad hoc questionnaire about physical activity and fitness self-assessment (T0, T1)  HADS (T1) | Depression  Anxiety | Reduction in the physical activity T1 vs T0 (p=0.034).  No difference in the self-assessment physical fitness T0 vs T1 (p=0.149).  The self-assessment of physical fitness explains anxiety in 30% and depression in 27% of PD.  No difference in depression (p=0.603) and anxiety (p=0.917) scores between PD living alone vs PD living with relatives. |
| Kumar et al. (2021) | India | 832 PD (570 M, 262 F) | NR | NR | Cross-sectional study | May 25-July 20, 2020 (during restrictions) | Survey | Depression  Anxiety  Sleep  QoL | 35.4% reported sleep problems.  Better quality sleep in subjects with adequate physical activity vs group of low physical activity at both times (p=0.01) and vs the group with reduced physical-activity during home confinement (p=0.02).  Higher prevalence of depression (p<0.001) and anxiety (p<0.001) in PD with new-onset/worsening sleep disturbance vs PD without sleep disturbances and PD with no-worsening sleep disturbances.  Most PD (54.2%) were not satisfied with their current QoL. |
| Luis-Martínez et al. (2021) | Italy | 12 PD (8 M, 4 F) | 69.5 (67-73.8)^b^ | 10.0 (8.0; 13.3)^b^ | Longitudinal study | T0: 2 weeks before March 11, 2020 (before restrictions)  T1: after May 4, 2020 (after restrictions) | ADL and IADL (T0, T1)  PDQ-39 (T0, T1) | QoL | No differences in ability to perform ADL and IADL score T0 vs T1 (p>0.05).  No differences in PDQ-39 score between T0 and T1 (p>0.05). |
| Sahin et al. (2021) | Turkey | 98 PD (52 M, 46 F)  577 other chronic neurological diseases  (225 M, 352 F) | PD:  65±11^a^  Other chronic neurological diseases  (49 ± 17)^a^ | NR | Case-control study | Data collected with reference to March-May, 2020 (during restrictions) | WHOQOL-BREF | QoL | PD reported lower scores in the social subscale vs other medical conditions (p=0.002).  No significant difference among the disease groups in WHOQOL-BREFs physical, mental, and environmental scores. |
| Saluja et al. (2021) | India | 64 PD (39 M, 25 F) | PD: 65 (55.25-69.75)b | 4 (2.5-7)^b^ | Cross-sectional study | June-September, 2020 (after restrictions) | NMSS  PDQ-8 (PD) | Depression  Sleep  QoL | Subjective worsening symptoms in 26 PD:  mood 42,3%, insomnia 34,6%, hypersomnolence in 7.7%.  Worse QoL was predicted by worsening of NMS (p=0.0005). |
| Silva-Batista et al. (2021) | Brazil | 478 PD (311 M, 167 F) | 67.3±9.5^a^ | 8.5±6.3^a^ | Cross-sectional study | May-June, 2020 (during COVID-19 pandemic) | PDQ-8 | QoL | Good QoL reported by 49.7% PD, no variation in QoL reported by 30.3 % PD. |
| Suzuky et al. (2021) | Japan | 100 PD (45 M, 55 F)  100 Caregivers (47 M, 53 F) | PD: 72.2±9.1^a^  Caregivers: 65.5±12.0^a^ | 5.8±4.4^a^ | Case-control study | June-December, 2020 with reference to after mid April 2020 (during restrictions) | HADS  PGIC scale (Only for PD)  SF-8 | Depression  Anxiety  Depression  Sleep  QoL | No differences in prevalence of depression (p=0.585) and anxiety (p=1.000) in PD vs Caregivers.  PD self-reported no change in mood (56%) and sleep (48%).  Physical function, role physical, general health, vitality and PCS scores were significantly lower in PD vs Caregivers (p<0.001). |
| Templeton et al. (2021) | USA | 28 PD (13 M, 15 F) | NR | 6.07^a^ | Cross-sectional study | Exact timing NR | Survey | QoL  Sleep | Most PD felt at their best after physical activity (67.86%), being in a comfortable environment (60.71%), at the beginning of the day.  Decreased duration of physical activity during the stay-at-home mandate (p=0.022).  Worsened one symptom in 82.14% of individuals.  Slight subjective worsening of sleep, mainly in females than males. |
| Krzysztoń et al. (2022) | Poland | 47 PD (30 M, 17 F) | 72.1±1.3^a^ | NR | Cross-sectional study | December 23, 2020-June 23, 2021 (during COVID-19 pandemic) | Survey | Depression  Anxiety  QoL | 9% of PD self-reported a worsening of depressive symptoms.  Depressive symptoms worsen as the feeling of being alone/isolated increases (p=0.017).  38% of PD self-reported anxiety.  Anxiety symptoms decrease for:  deterioration of contacts (p=0.035);  and feelings of isolation (p=0.007).  QoL decrease for:  deterioration of contacts (p=0.022);  feelings of isolation (p=0.009). |
| Montanaro et al. (2022) | Italy | 100 PD (60 M, 40 F)  60  Caregivers (21 M, 39 F) | PD:  62.4±9.0^a^  Caregivers: 62.1±9.2^a^ | 13.4±4.6^a^ | Case-control studies | T0: April-May, 2020 (during restrictions)  T1: June-August 2020 (after restrictions) | HADS (T0: PD, Caregivers; T1: PD)    Ad hoc questionnaire (T0: PD) | Depression  Anxiety  Sleep | Worse depression score in PD (35%) vs Caregivers (21.7%) at T0 (p=0.001).  39% PD and 40% Caregivers presented anxiety at T0.  PD:  No differences in prevalence of depression in PD T0 (35%) vs T1 (34.1%; p= 0.807).  Decreased prevalence of anxiety in PD T1 (30,6%) vs T0 (39%; p=0.023).  Worse anxiety score in PD treated with different types of therapy (p= 0.004).  From the subjective report most PD referred  no difficulty falling asleep (54%) at T0. |

^a^ data presented as the means with standard deviation (SD)

^b^ data presented as the medians with interquartile range (IQR)

Legend: ADL, Activities of Daily Living; BAI-II, Beck Anxiety Inventory II; BDI, Beck Depression Inventory; BDI-II, Beck Depression Inventory-II, CG, control group; DASS-21, Depression, Anxiety, and Stress Scale–21; F, female; GAD-7, 7-item Generalized Anxiety Disorder; GDS, Geriatric Depression Scale; HADS, Hospital Anxiety and Depression Scale; HC, healthy controls; IADL, Instrumental Activities of Daily Living; M, male; ISI, 7-item Insomnia Severity Index; MDS-UPDRS, Movement Disorders Society-Unified Parkinson disease rating Scale; NMS, Non-Motor Symptoms; NMSS, Non-Motor Symptoms Scale; NR, not reported; OR-PAS, 12 item Parkinson Anxiety Scale; PAS, Parkinson Anxiety Scale; PCS, Physical Component Summary; PD, Parkinson’s disease patients; PDQ-8, 8-items Parkinson’s Disease Questionnaire; PDQ-39, 39-items Parkinson’s Disease Questionnaire; PGI-I, Patient’s Global Impression-Improvement; PGIC, Patient Global Impression of Change; PHQ-9, 9-item Patient Health Questionnaire; PRO-PD, Patient-Reported Outcomes in Parkinson’s Disease, PROMIS, Patient-Reported Outcomes Measurement Information System; PSQI, Pittsburgh Sleep Quality Index; QoL, Quality of Life; RBD, Rapid eye movement sleep behaviour disorders; SE-ADL, The Schwab and England Activities of Daily Living scale; SF-8, 8-item Short Form; STAI, State-Trait Anxiety Inventory; VS, versus; WHOQOL-BREF, World Health Organization Quality of Life short form; YR, years.
